# Chemical Transformation Induced Core–Shell Ni_2_P@Fe_2_P Heterostructures toward Efficient Electrocatalytic Oxygen Evolution

**DOI:** 10.3390/nano12183153

**Published:** 2022-09-11

**Authors:** Huijun Song, Jingjing Li, Guan Sheng, Ruilian Yin, Yanghang Fang, Shigui Zhong, Juan Luo, Zhi Wang, Ahmad Azmin Mohamad, Wei Shao

**Affiliations:** 1 State Key Laboratory Breeding Base of Green Chemistry Synthesis Technology, College of Chemical Engineering, Zhejiang University of Technology, Hangzhou 310014, China; 2 School of Materials and Mineral Resources Engineering, University Sains Malaysia, Nibong Tebal 14300, Malaysia

**Keywords:** Ni_2_P@Fe_2_P, heterostructures, oxygen evolution reaction

## Abstract

The oxygen evolution reaction (OER) is a crucial reaction in water splitting, metal–air batteries, and other electrochemical conversion technologies. Rationally designed catalysts with rich active sites and high intrinsic activity have been considered as a hopeful strategy to address the sluggish kinetics for OER. However, constructing such active sites in non-noble catalysts still faces grand challenges. To this end, we fabricate a Ni_2_P@Fe_2_P core–shell structure with outperforming performance toward OER via chemical transformation of rationally designed Ni-MOF hybrid nanosheets. Specifically, the Ni-MOF nanosheets and their supported Fe-based nanomaterials were in situ transformed into porous Ni_2_P@Fe_2_P core–shell nanosheets composed of Ni_2_P and Fe_2_P nanodomains in homogenous dispersion via a phosphorization process. When employed as the OER electrocatalyst, the Ni_2_P@Fe_2_P core–shell nanosheets exhibits excellent OER performance, with a low overpotential of 238/247 mV to drive 50/100 mA cm^−2^, a small Tafel slope of 32.91 mV dec^−1^, as well as outstanding durability, which could be mainly ascribed to the strong electronic interaction between Ni_2_P and Fe_2_P nanodomains stabilizing more Ni and Fe atoms with higher valence. These high-valence metal sites promote the generation of high-active Ni/FeOOH to enhance OER activity.

## 1. Introduction

Developing sustainable and clean energy is crucial for satisfying the ever-increasing energy demand and existing environmental problems [1,2,3]. However, the intermittency in generated energy sources, primarily through solar and wind, impedes the storage of produced energy, owing to limited grid-scale battery capacity. Electrochemical water splitting has great potential to utilize such intermittent energy to generate clean hydrogen sources. However, the hydrogen evolution reaction at the cathode is seriously limited by the sluggish kinetics in the OER (oxygen evolution reaction) at the anode. Thus, it is urgent to explore active electrocatalysts with high oxygen evolution reaction (OER) performance to achieve the industrial utility of water splitting for hydrogen production.

Until now, considerable efforts have been made to explore Earth-abundant and high-efficiency transition metal-based OER catalysts, including layered double hydroxides (LDHs) [4], metal oxides [5], phosphides [6], selenides [7], sulfides [8], nitrides [9], and so on. Among them, transition-metal phosphides (TMPs) have been considered as promising candidates for alkaline OER catalysis because of their good electrical conductivity with metalloid characteristics and remarkable durability in working conditions with strong alkaline electrolytes and theoretically outstanding electrochemical catalytic behaviors [10,11,12,13,14]. During the alkaline OER, the metal-p bond could promote the formation of metal oxyhydroxides with rich defects and low-crystalline properties. The TMPs could be transformed into TMPs/MO_x_ with a unique core–shell structure. The TMP core as conductive support could facilitate the rapid and effective electron transfer process and synergistically enhance the MO_x_ shell as species demonstrating higher electrochemical performance [15,16]. Although these TMP-based electrocatalysts have been proved to have great potential for the OER, their activities are still not enough to achieve the requirements of industrial applications with large current density (≥500 mA cm^−2^) at low overpotentials (<300 mV) [17]. For further improvement in the catalytic behaviors of TMP-based catalysis, heterostructure engineering has been considered as an effective strategy and widely carried out. The heterostructure can be extensively fabricated through the hybridization of various transition-metal electrocatalysts. It can merge the structural advantages of each component and generate abundant active sites and electronic reconfigured interfaces, which collectively accelerate the reaction kinetics and, thus, modify the catalytic performance of nanocomposites [18,19,20]. For example, Huang regulated the electronic configuration of Ni and N atoms around the Fermi level to boost overall water splitting by constructing the heterointerface of Ni_3_N@2M-MoS_2_ [21]. Du modulated the local structures and electronic environments via constructing Cu@CeO_2_ nanotube with the deposition of NiFeCr hydroxide, leading to catalysts with sufficient active sites, quick oxygen diffusion, and releasement; the d–f orbital coupling for great promoted electron transfer and, therefore, demonstrating enhanced OER performance [22]. However, constructing catalytically active heterostructures with novel composition and architecture still remains poorly developed due to the synthetic challenge.

Considering the above discussion, herein, a simple strategy was proposed to prepare Ni_2_P@Fe_2_P core–shell nanosheets as high active catalysts for OER by the chemical transformation of rationally fabricated Ni-MOF hybrid nanosheets. The obtained Ni_2_P@Fe_2_P 2D hieratic structure was composed of homogeneous dispersion of Fe_2_P and Ni_2_P nanodomains, which induces the synergistic effect of different components, affording the Ni_2_P@Fe_2_P nanosheets with more stabilized high-valence metal active sites to promote the generation of high-active Ni/FeOOH during the OER process and result in outperforming OER performance. As expected, the designed Ni_2_P@Fe_2_P nanosheets demonstrate ultralow overpotentials of 210/247 mV at 10/100 mA cm^−2^ and afford more than 36 h durability with negligible decay in OER under multi-current densities.

## 2. Materials and Methods

The experimental details, including the Materials and Methods, are listed in the Supplementary Materials.

## 3. Results and Discussion

**Figure 1:**
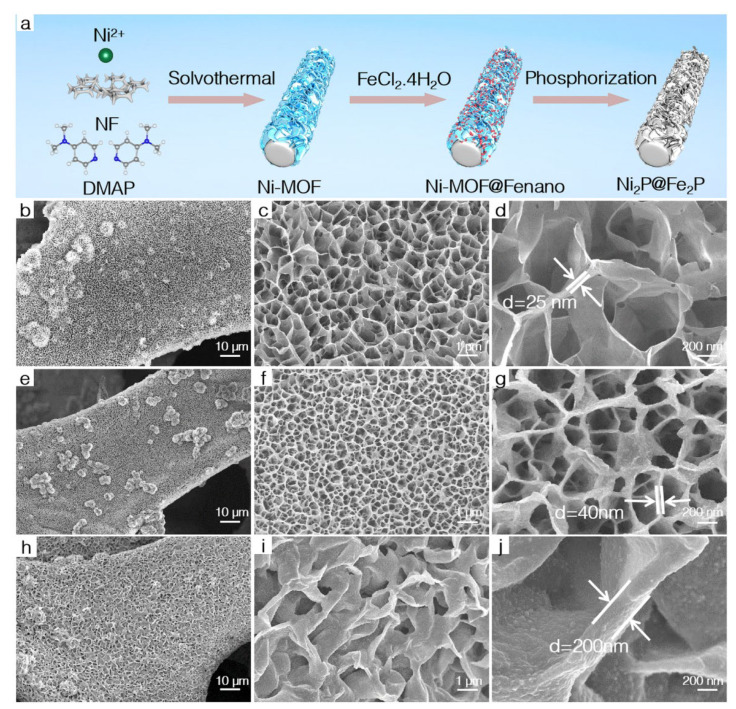
(**a**) Schematic illustration for the synthesis of the Ni_2_P@Fe_2_P. SEM images of Ni-MOF (**b**–**d**), Ni-MOF@Fenano (**e**–**g**) and Ni_2_P@Fe_2_P (**h**–**j**).

Figure 1a schematically illustrates the fabrication of NF-supported hierarchical 2D core–shell Ni_2_P@Fe_2_P nanosheets. First, the novel 2D Ni-MOF nanosheets were synthesized on NF using DMAP as organic ligands coordinated with nickel salts. Subsequently, the as-prepared Ni-MOF nanoarray was soaked in FeCl_2_·4H_2_O ethanol solution at 90 °C for 15 h and the Ni-MOF uniformly covered with Fe-based nanoparticles on the surface was obtained, followed by the phosphorization treatment with NaH_2_PO_4_·2H_2_O as a phosphorus source to prepare the hierarchical 2D core–shell Ni_2_P@Fe_2_P nanosheets. As shown in Figure 1b–d, in situ grown self-supported Ni-MOF nanosheets are highly dispersive and vertically stacked on the Ni foam. In addition, the lateral size and thickness of the nanosheets are several hundred nanometers and about 25 nm, respectively. After soaking treatment in the FeCl_2_·4H_2_O ethanol solution, the surface of obtained Ni-MOF@Fenano nanosheets was covered with some tiny nanoparticles (Figure 1e,f). In addition, the thickness of the nanosheets increased to 40 nm. The corresponding energy-dispersive X-ray spectroscopy (EDS) mapping images (Figure S1) indicate that the Ni and Fe elements are mainly dispersed in the inner nanosheets and out of tiny nanoparticles, suggesting Ni-MOF@Fenano with a core–shell structure. The EDS spectrum (Figure S2) reveals that the ratio of Fe/Ni is about 0.096. After chemical transformation by thermal phosphorization at 300 °C for 2 h with NaH_2_PO_4_·2H_2_O, it is obvious that the obtained Ni_2_P@Fe_2_P basically inherited the overall morphology of Fe-based nanoparticle-decorated Ni-MOF nanosheets. The pure Ni_2_P nanosheets and Fe_2_P supported on the NF were also fabricated as reference. Figure 1e–g demonstrate that the Ni-MOF-derived Ni_2_P nanosheets were vertically stacked on the NF and plenty of large holes could be obviously found in Ni_2_P nanosheets (Figure S3), while the synthesized Fe_2_P exhibited irregular nano bulks (Figure S4). Therefore, we can infer that during the thermal phosphorization of the Ni-MOF@Fenano materials, the Fenano shell and Ni-MOF core were etched by PH_3_ and then transformed into core–shell structured Ni_2_P@Fe_2_P nanosheets and the Fe_2_P shell can act as a protecting layer, preventing the structure collapse of Ni_2_P nanosheets.

**Figure 2:**
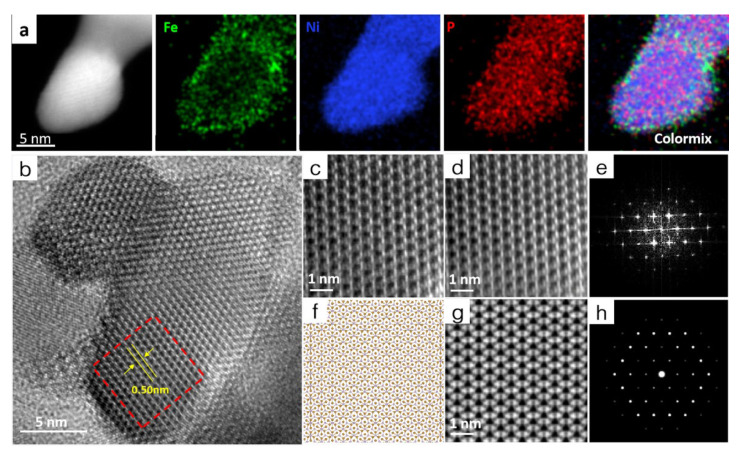
(**a**) HAADF-STEM and the corresponding EDS mapping images of Ni_2_P@Fe_2_P. (**b**) High-magnification TEM image of Ni_2_P@Fe_2_P heterostructure. (**c**) Select-area HRTEM view of the crystalline region of Ni_2_P. (**d**) Denoised image of Figure 2c. (**f**) The crystal structure model of Ni_2_P observed along the [001] axis. (**g**) The projected potential of Ni_2_P observing along the [001] axis. (**e**) FFT of Figure 2d and (**h**) simulated FFT of the crystal structure model of Ni_2_P observed along the [001] axis.

**Figure 3:**
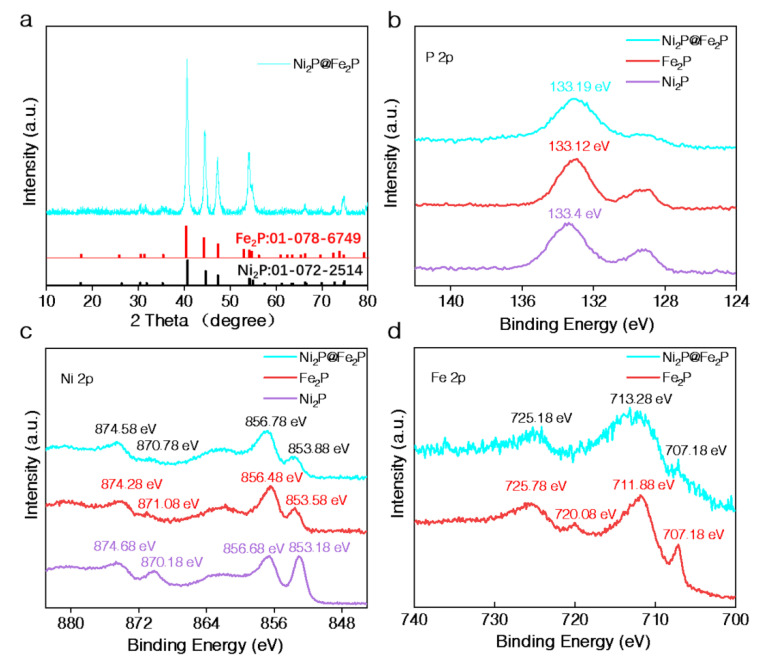
(**a**) XRD pattern of Ni_2_P@Fe_2_P. (**b**) High-resolution P 2p XPS spectra of the Ni_2_P@Fe_2_P, Fe_2_P and Ni_2_P. (**c**) High-resolution Ni 2p XPS spectra of the Ni_2_P@Fe_2_P, Fe_2_P and Ni_2_P. (**d**) High-resolution Fe 2p XPS spectra of the Ni_2_P@Fe_2_P and Fe_2_P.

High-angle annular dark-field scanning transmission electron microscopy (HAADF-STEM) was used to detect the element composition and dispersion in the Ni_2_P@Fe_2_P. The HAADF image in Figure S5 shows that the core–shell Ni_2_P@Fe_2_P nanosheets are composed of tiny nanoparticles. The EDS mapping images (Figure 2a and Figure S5) of the Ni_2_P@Fe_2_P clearly state that the Ni and Fe elements are mainly distributed in the core and shell of Ni_2_P@Fe_2_P, respectively, while the P elements are homogeneously distributed over the entire Ni_2_P@Fe_2_P. A similar ratio of Fe/Ni to that for Ni-MOF@Fenano was determined by the EDS spectrum (Figure S6). XRD test was firstly carried out to determine the crystal phases of as-prepared Ni_2_P@Fe_2_P, Fe_2_P, and Ni_2_P. As shown in Figure 3a, all the diffraction peaks of the Ni_2_P@Fe_2_P match well with Fe_2_P (JCPDS No. 01-078-6794) or Ni_2_P (JCPDS No. 01-072-2514), suggesting a similar crystal structure of Fe_2_P and Ni_2_P nanodomains in Ni_2_P@Fe_2_P nanosheets. In addition, the Fe_2_P demonstrates similar diffraction peaks with that of Ni_2_P@Fe_2_P, while two additional peaks (located at 38.5° and 49.1°) indexed to Ni_12_P_5_ (JCPDS No. 22-1190) could be found in as-prepared Ni_2_P samples (Figure S8). The HRTEM was further conducted for the atomic-scale structural identification of Ni_2_P@Fe_2_P nanosheets. Figure 2b shows a HRTEM image of Ni_2_P@Fe_2_P nanosheets. The selected area (marked with a red rectangle in Figure 2b) HRTEM image (Figure 2c) and its denoised image (Figure 2d) unravel the {010} planes where the lattice spacing is 5.0 Å, affirming its identity from crystallography since it matches well with the crystalline model of Ni_2_P (or Fe_2_P, because of their same crystal structure) observed along the [001] axis (Figure 2f) and the corresponding projected potential (Figure 2g). The corresponding Fast Fourier transform pattern (Figure 2e) in Figure 2d also fits well with the simulated corresponding FFT (Figure 2h). In conclusion, the HRTEM results reveal that the Ni_2_P@Fe_2_P 2D hieratic structure is composed of homogeneous dispersion of Fe_2_P and Ni_2_P nanodomains.

Heterostructure engineering has been reported to be an effective strategy to modulate the electronic structure of active sites and, therefore, effectively adjusts the catalytic behaviors of the catalysts [18,23,24]. Hence, an X-ray photoelectron spectroscopy (XPS) test was conducted to investigate the surface electronic configuration and chemical composition of prepared samples. The survey spectra (Figure S7) confirm the coexistence of Ni, Fe, P, and O elements in Ni_2_P@Fe_2_P and Fe_2_P samples, while the prepared Ni_2_P is mainly composed of Ni, P, and O elements, which suggests the successful decoration of Fe-based nanomaterials on the Ni-MOF nanosheets. The Ni element in Fe_2_P samples may be ascribed to the generation of Ni_2_P resulting from the corrosion of Ni foam by PH_3_. The existence of O could originate from the surface oxidation of samples in the air or unavoidable adsorbed oxygen species. In the high-resolution P 2p spectra, the Fe_2_P and Ni_2_P@Fe_2_P exhibit lower binding energy than that of Ni_2_P (Figure 3b). In the high-resolution Ni 2p spectra of all samples (Figure 3c), two peaks located at about 856 eV and 874 eV with satellite peaks can be attributed to the Ni^2+^ 2p 3/2 and Ni^2+^ 2p 1/2 of Ni^2+^ in surface oxide [25]. In addition, a single peak of about 853 eV could be assigned to Ni-P species [26]. The binding energy of Ni-P is lower than that of Ni-O due to the weaker electronegatively of P, which means difficulty for electrons to dissociate from the metal ion. It is noted that the peaks for Ni-O and Ni-P species of Ni_2_P@Fe_2_P and Fe_2_P exhibit large and small positive shifts, respectively, compared with those observed from the Ni_2_P spectrum. A similar positive shift in the peaks for Fe-O and Fe-P species of Ni_2_P@Fe_2_P is evident compared with those observed from the Fe_2_P spectrum (Figure 3d). Such positive shifts indicate the generation of more Ni and Fe atoms with higher valence, which imply the strong electronic interaction between Ni_2_P and Fe_2_P in Ni_2_P@Fe_2_P nanosheets. In addition, stabilized metals with higher valence have been widely considered with outperforming performance for OER, because they could enhance the chemisorption of OH^−^ and promote the in situ generation of MOOH as high-active sites (M represents metal) through nucleophilic attack during OER [27,28]. Therefore, the as-synthesized biphasic Ni_2_P@Fe_2_P nanosheets demonstrate great potential to evoke synergistic electrocatalysis for OER.

**Figure 4:**
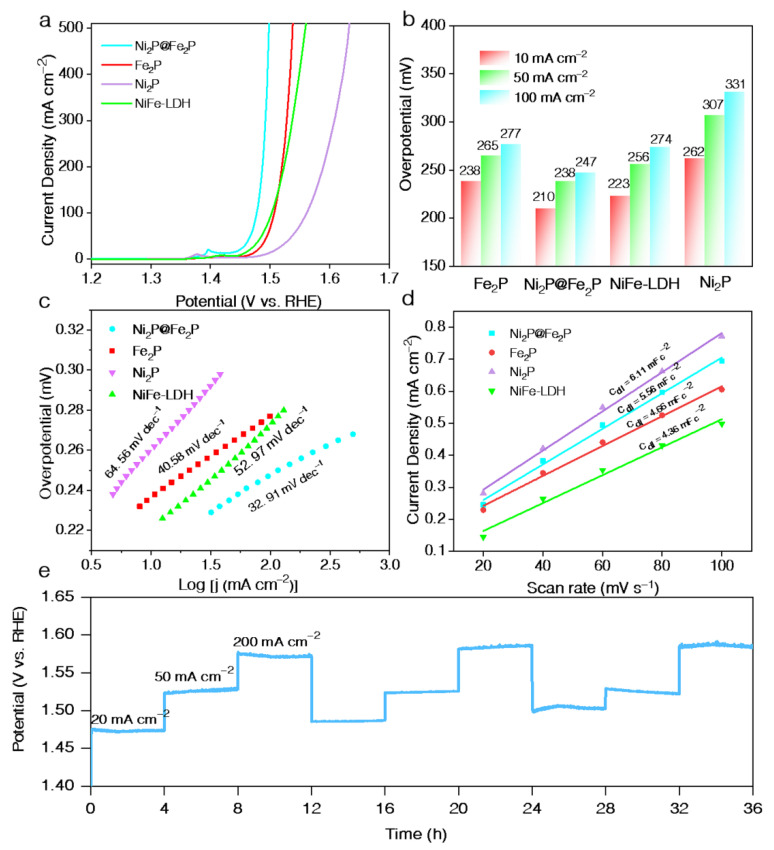
(**a**) LSV curves of Fe_2_P, Ni_2_P@Fe_2_P, NiFe-LDH and Ni_2_P. (**b**) Comparison of the overpotentials at 10 mA cm^−2^, 50 mA cm^−2^ and 100 mA cm^−2^ for Fe_2_P, Ni_2_P@Fe_2_P, NiFe-LDH and Ni_2_P. (**c**) Tafel curves of Fe_2_P, Ni_2_P@Fe_2_P, NiFe-LDH and Ni_2_P. (**d**) C_dl_ values of Ni_2_P, NiFe-LDH, Ni_2_P@Fe_2_P and Fe_2_P. (**e**) Durability test of Ni_2_P@Fe_2_P at constant 20 mA cm^−2^, 50 mA cm^−2^ and 200 mA cm^−2^.

The OER performance was evaluated using a three-electrode system in 1 M KOH. The NF supported NiFe-LDH commercial RuO_2_ and IrO_2_ deposited on the NF were also assessed as references. SEM images in Figure S9 show that the prepared NiFe-LDH is composed of plenty of nanowires and nanosheets. Figure 4a and Figure S10 display the polarization curves (corrected with iR loses) for all samples. The Ni_2_P@Fe_2_P catalyst demonstrates the highest activity, requires a small overpotential (*η*) of 210 mV to achieve a current density of 10 mA cm^−2^, outperforming Fe_2_P (238 mV), Ni_2_P (262 mV), NiFe-LDH (223 mV), RuO_2_ (292 mV), IrO_2_ (337 mV), and pure NF (390 mV) (Figure 4b). Furthermore, the Ni_2_P@Fe_2_P can reach a large current density of 50 and 100 mA cm^−2^ at the overpotential of 238/247 mV, which is comparable with the most reported active transition-metal-based OER electrocatalysts (Table S1) [28,29,30,31,32,33,34,35,36,37,38,39,40,41,42,43]. Of note, the peaks around 1.36–1.41 V (vs. RHE) in Figure 4a are corresponding with the oxidation of Ni species [44,45]. Among them, the redox peak of Ni_2_P@Fe_2_P nanosheets shows a positive shift compared with that of Ni_2_P nanosheets, suggesting the formation of Ni^4+^ of Ni_2_P@Fe_2_P during OER, which has the modulated d-band center and reduced the adsorption energy of oxygenated intermediates on the surface of the catalyst. In addition, the increased Ni^4+^ content will lead to greater Ni-O covalency during OER and, thus, greater oxyl character, which could directly correlate with enhanced activity of the catalyst in promoting OER [46,47]. The corresponding Tafel plots (Figure 4c) show a decreasing trend in Tafel slope from Ni_2_P (64.56 mV dec^−1^), NiFe-LDH (52.97 mV dec^−1^), Fe_2_P (40.58 mV dec^−1^) to Fe_2_P@Ni_2_P (32.91 mV dec^−1^), demonstrating the faster OER kinetics for the Fe_2_P@Ni_2_P electrode, which may be ascribed to the modulated electronic structure of metal active sites according to the LSV and XPS results. To gain further insights into the superior OER activity of Fe_2_P@Ni_2_P electrode catalysts, cyclic voltammetry (CV) curves with different scan rates were collected to analyze electrochemically double-layer capacitance (C_dl_), which is proportional to electrochemically active surface area (ECSA) (Figure S11). As displayed in Figure 4d, the Ni_2_P nanosheets have the highest value of C_dl_ of 6.11 mF cm^−2^, which is larger than that of Ni_2_P@Fe_2_P (5.56 mF cm^−2^) and Fe_2_P (4.66 mF cm^−2^), indicating that the Ni_2_P nanosheets enhance more accessible active sites in the core–shell Ni_2_P@Fe_2_P for the OER. Notably, the lowest OER activity of Ni_2_P indicates that the intrinsic activity rather than the ECSA of the samples is the key to OER activity. Furthermore, the chronopotentiometry experiments without iR compensation were conducted to explore the durability. Figure 4e presents the chronopotentiometry response of Ni_2_P@Fe_2_P at multicurrent densities. A negligible activity degradation is observed for Ni_2_P@Fe_2_P after a 36 h test. In addition, the stability test of IrO_2_ at 10 mA cm^−2^ demonstrates obvious activity degradation after a 5 h test (Figure S12a). This suggests the superb durability of the core–shell Ni_2_P@Fe_2_P for OER. The EIS characterizations were also tested and the Nyquist plot (Figure S12b) demonstrates that the Ni_2_P@Fe_2_P has the smallest Rct, suggesting its faster electron transport during OER, thus, promising a higher OER activity.

Previous reports have demonstrated that many transition-metal-based OER catalysts undergo substantial reconstruction to form mixed-metal oxyhydroxides [48,49,50]. Hence, the structure information of the catalysts after the LSV test was investigated to identify the intrinsic origins of the excellent OER performance. SEM and TEM images (Figure S13) show that the morphology of Ni_2_P@Fe_2_P demonstrated obvious changes; plenty of newly formed small nanosheets on the surface of Ni_2_P@Fe_2_P nanosheets were observed. The corresponding HAADF STEM mapping (Figure S14) revealed that the newly formed small nanosheets are mainly composed of Fe, Ni, and O elements, while the inner nanosheets are mainly composed of Fe, Ni, and P elements, indicating significant surface structure conversion of Ni_2_P@Fe_2_P nanosheets during the OER process. In addition, a newly formed Raman band (Figure S15) located at about 558 cm^−1^ was observed for Ni_2_P@Fe_2_P and this new band is likely to be ascribed to Fe/Ni-O vibrations in NiFeOOH [26,49]. Therefore, we can infer that the surface of Ni_2_P@Fe_2_P was partially transformed into NiFeOOH nanosheets during the OER process and resulted in hierarchically core–shell structured nanosheets. The XPS spectra of Ni_2_P@Fe_2_P after the LSV test were also conducted to investigate the evolution of elemental composition and valence. The disappearance of characteristic peaks for metal-p bond in the Ni and Fe fine spectra further demonstrate the surface transformation of Ni_2_P@Fe_2_P during the OER process and the positive shift in the binding energy for Fe-O and Ni-O species may be ascribed to the formed NiFeOOH on the surface of Ni_2_P@Fe_2_P (Figure S16). Therefore, we can infer that the interactions between Ni_2_P and Fe_2_P in catalysts promote its surface transformation during the OER process and the in situ formatted metal oxyhydroxides from the TM-based electrocatalysts usually are considered with high activity toward OER.

## 4. Conclusions

In summary, a well-defined Ni_2_P@Fe_2_P nanosheet structure as an excellent electrocatalyst for OER was successfully built through the chemical transformation of Ni-MOF hybrid nanosheets. Benefiting from their heterostructure nanosheet affording the synergetic effect of the two components, the reduced overpotential was achieved through stabilized high-valence metal sites promoting the formation of Ni/FeOOH. The resultant Ni_2_P@Fe_2_P nanosheets electrocatalyst exhibits outperforming OER performance with a low overpotential of 210 mV at 10 mA cm^−2^, a Tafel slope of 32.91 mV dec^−1^, and excellent durability in 36 h of OER test.

## Data Availability

The data that support the findings of this study are available from the corresponding authors upon reasonable request.

## Supplementary Materials

The following supporting information can be downloaded at: https://www.mdpi.com/article/10.3390/nano12183153/s1, Figure S1: HAADF STEM and corresponding EDS mapping images of Ni-MOF@Fenano. Figure S2: EDS spectrum of Fenano. Figure S3: (a,b) SEM images of Ni_2_P. Figure S4: SEM images of Fenano (a,b) and Fe_2_P (c,d). Figure S5: HAADF STEM and corresponding EDS mapping images of Ni_2_P@Fe_2_P. Figure S6: EDS spectrum of as-prepared Ni_2_P@Fe_2_P. Figure S7: XPS survey spectra of the Ni_2_P, Ni_2_P@Fe_2_P and Fe_2_P.Figure S8: XRD patterns of (a) Fe_2_P and (b) Ni_2_P. Figure S: (a–c) SEM images of LDH with different magnifications. Figure S10: LSV curves of IrO_2_, RuO_2_ and Ni foam. Figure S11: CV curves of (a) Fe_2_P, (b) NiFe-LDH, (c) Ni_2_P@Fe_2_P, and(d) Ni_2_P acquired at various scan rates. Figure S12: Durability test of Ir_2_O@10 mA cm^−2^. (b) Nyquist plots. Figure S13: (a,b) SEM images of Ni_2_P@Fe_2_P after LSV test. (c) TEM image of Ni_2_P@Fe_2_P. (d) TEM image of Ni_2_P@Fe_2_P after LSV test. Figure S14: HAADF STEM and corresponding EDS mapping images of Ni_2_P@Fe_2_P after LSV test. Figure S15: Raman spectra of Ni_2_P@Fe_2_P before and after LSV test. Figure S16: High-resolution XPS spectra of (a) Ni 2p, (b) Fe 2p and (c) Ni_2_P@Fe_2_P after LSV test. Table S1: Comparison of OER performances between Ni_2_P@Fe_2_P electrode and recently reported electrocatalysts in alkaline solution. References [28,29,30,31,32,33,34,35,36,37,38,39,40,41,42,43] is cited in the Supplementary Materials.
