# Supplementary material for: Chemical Transformation Induced Core–Shell Ni2P@Fe2P Heterostructures toward Efficient Electrocatalytic Oxygen Evolution
Source: Nanomaterials (Basel). 2022 Sep 11;12(18):3153. doi: 10.3390/nano12183153 (PMC9503841; doi:10.3390/nano12183153)
Supplement: Supplementary file 1 [file nanomaterials-12-03153-s001.zip › nanomaterials-1902864-supplementary .pdf]

nanomaterials

Supplementary Material

Chemical Transformation induced Core-shell Ni_2P@Fe_2P Heterostructures toward Efficient Electrocatalytic Oxygen Evolution

Huijun Song [1,†], Jingjing Li [1,†], Guan Sheng [2,†], Ruilian Yin [1], Yanghang Fang [1], Shigui Zhong, Juan Luo [1], Zhi Wang[1], Ahmad Azmin Mohamad [2], and Wei Shao [1,*]

1 State Key Laboratory Breeding Base of Green Chemistry Synthesis Technology, College of Chemical Engineering, Zhejiang University of Technology, Hangzhou 310014, China
2 School of Materials and Mineral Resources Engineering, University Sains Malaysia, Nibong Tebal 14300, Malaysia
* Correspondence: weishao@zjut.edu.cn
† These authors have contributed equally to this work.

1. Materials and Methods

1.1. Reagents and Chemicals

Nickel nitrate hexahydrate ($Ni(NO_3)_2·6H_2O$), iron dichloride ($FeCl_2·4H_2O$), ferric trichloride ($FeCl_3·6H_2O$), Sodium dihydrogen phosphate dihydrate ($NaH_2PO_4·2H_2O$), and acetone were analysis reagents (AR) and obtained from Aladdin Biochemical Technology Co. Ltd. (Shanghai, China). Ethanol, methanol, and hydrogen chloride (HCl) were of AR grade and purchased from Shanghai Macklin Biochemical Technology Co., Ltd. (Shanghai, China**).** 4-Dimethylaminopyridine (DMAP), urea ($CO(NH_2)_2$, Iridium dioxide (IrO_2) and Ruthenium oxide (RuO_2) were provided by Sigma-Aldrich (St. Louis, MO, USA).

1.2. Methods

To synthesize Ni foam-supported Ni-MOF nanosheet, a piece of pretreated Ni foam (NF) was firstly cut into 1 cm× 3 cm, treated with acetone, diluted hydrochloric acid and ethanol in sequence under sonication for 0.5 h. Then the pretreated Ni foam and a solution containing 1 mmol $Ni(NO_3)_2·6H_2O$, 2 mmol DMAP and 10 mL of methanol were put into a Teflon autoclave. Subsequently, the autoclave was maintained at 100 °C for 4 h. Finally, the NF supported Ni-MOF nanosheet was rinsed with methanol and vacuum dried at 40 °C for 24 h.

For the synthesis of core-shell Ni-MOF@Fe-based nanomaterials (Ni-MOF@Fenano), $FeCl_2·4H_2O$ (80 mg) was dissolved in 20 mL ethanol using ultrasound for 5 min and then transferred into a 25 mL glass vial. Subsequently, the as-synthesized Ni-MOF nanosheet was vertically put into the above vial and reacted for 24 h at 90 °C. The formed Ni-MOF@Fenano was washed with ethanol (30 mL) to remove unreacted precursors and then dried naturally. The NF supported Fe-based nanomaterials (Fenano) were also prepared by similar methods except by replacing Ni-MOF with pure pretreated NF.

For the synthesis of core-shell Ni_2P@Fe_2P nanosheets, the as-prepared Ni-MOF@Fenano and 0.5 g $NaH_2PO_4·2H_2O$ were put at the downstream and upstream sides of the tubular furnace, respectively. Then the tubular furnace was set to 300 °C for 2 h with a heating ramp of 2 °C min^{-1} under flowing N_2 to achieve the final product (Ni_2P@Fe_2P). For comparison, Ni_2P nanosheets and Fe_2P bulks were obtained through phosphating Ni-MOF nanosheets and Fenano precursors under the same process.

The synthesis of Ni foam supported NiFe-LDH was performed according to literature procedures with some modifications [1]. Typically, 0.5 mmol $Ni(NO_3)_2·6H_2O$, 0.5 mmol $FeCl_3·6H_2O$ and 4 mmol $CO(NH_2)_2$ were dissolved in 35 mL of DI water in a 50-mL Teflon-lined flask. After a piece of pre-treated NF was added to the above solution, the Teflon-lined flasks were sealed in autoclaves and then kept under static conditions in a preheated oven at 125 °C for 12 h. Finally, the sample was taken out, washed with DI water, and dried in a vacuum oven.

1.3. Materials Characterizations

The morphologies of the synthesized materials were analyzed by scanning electron microscopy ZEISS-G500 (ZEISS, Jena, Germany). The high-resolution TEM (HRTEM), scanning transmission electron microscope (STEM) and elemental mapping images were obtained via an FEI 80- 300kV Cs corrected on Titan Themis Z (Thermo Fisher Scientific, Waltham, MA, USA). The Raman spectra of all synthesized samples were obtained using a HORIBA Jobin–Yvon

Lab-Ram ARAMIS (Horiba, Kyoto, Japan). X-ray diffraction (XRD) patterns of the as-prepared products were obtained through a D8Advance (Bruker, Billerica, MA, USA). The surface properties of all samples were characterized using X-ray photoelectron spectroscopy (XPS) ESCALAB 250, (Thermo Fisher Scientific, Waltham, MA, USA).

1.4. Electrochemical Measurements

All the electrochemical performance was evaluated in 1.0 M KOH with a standard three-electrode system via CHI 760E electrochemical workstation. The NF supported catalysts were used as the working electrode, while the saturated Ag/AgCl electrode and Pt wire were served as a reference electrode and a counter electrode, respectively. The measured potentials were calibrated to a reversible hydrogen electrode (RHE) according to the formula of $E_{RHE} = E_{Ag/AgCl} + 0.059 \times pH + 0.197$ (V). Linear sweep voltammetry (LSV) measures corrected with 95% iR loses were performed with a scan rate of 0.2 mV s^{-1}. Multicurrent densities were used for durability test. The double-layer capacitance (C_{dl}) was calculated based on the cyclic voltammograms (CVs) test with different scan rates over a non-faradaic potential range of 0.1–0.2 V ($E_{Ag/AgCl}$) in 1.0 M KOH.

2. Supplementary Figures and Table.

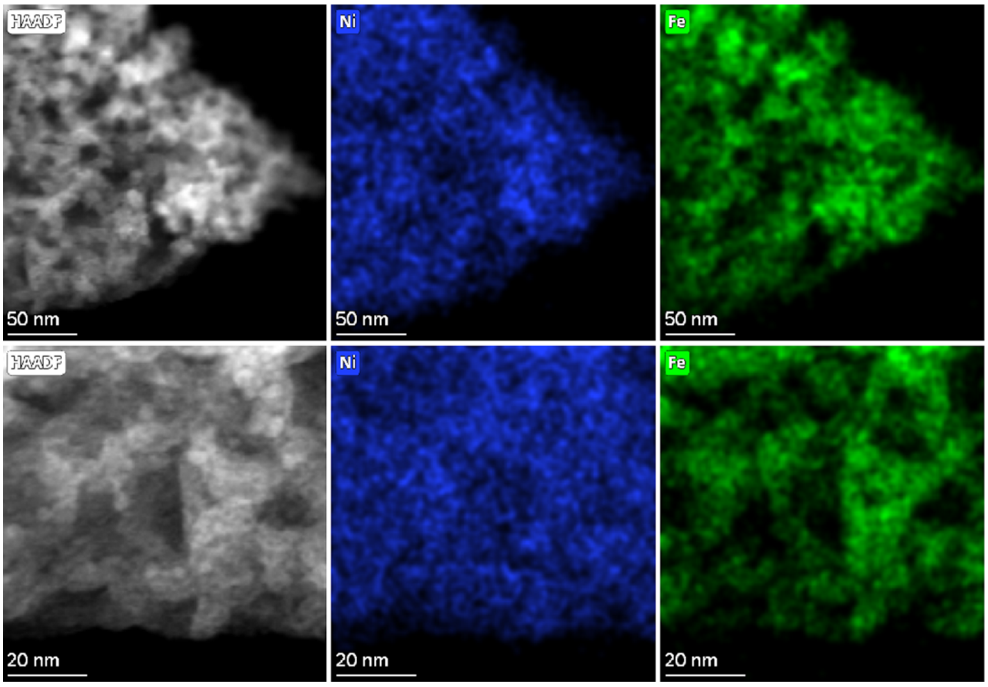

Figure S1. HAADF STEM and corresponding EDS mapping images of Ni-MOF@Fenano.

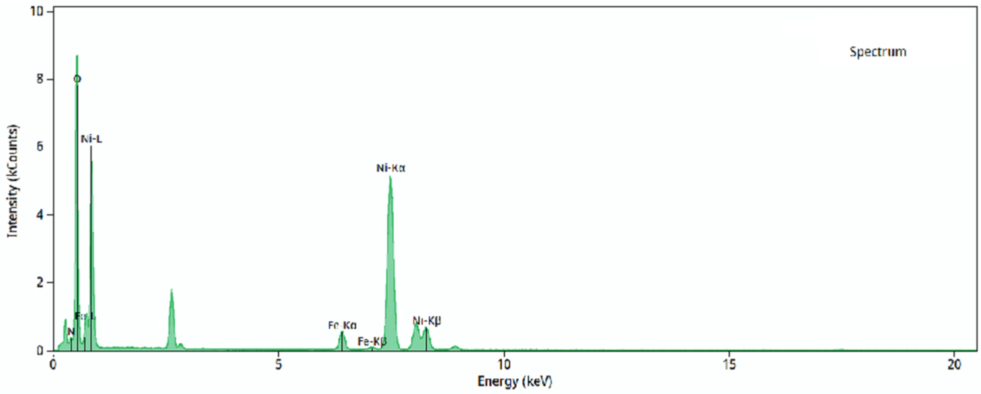

Z	Element	Family	Atomic Fraction (%)	Atomic Error (%)	Mass Fraction (%)	Mass Error (%)	Fit error (%)
7	N	K	3.74	0.76	1.72	0.19	0.47
8	O	K	62.02	17.75	32.58	7.45	1.04
26	Fe	K	3.00	0.73	5.49	0.95	0.64
28	Ni	K	31.24	7.87	60.21	11.05	0.13

Figure S2. EDS spectrum of Fenano.

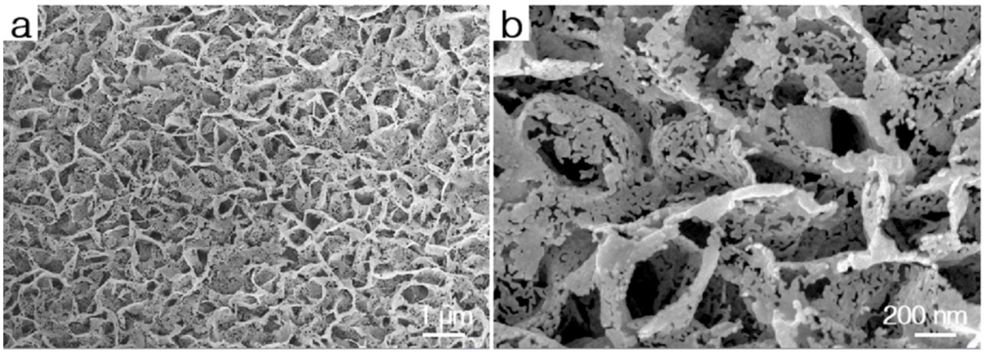

Figure S3. (**a**,**b**) SEM images of Ni_2P.

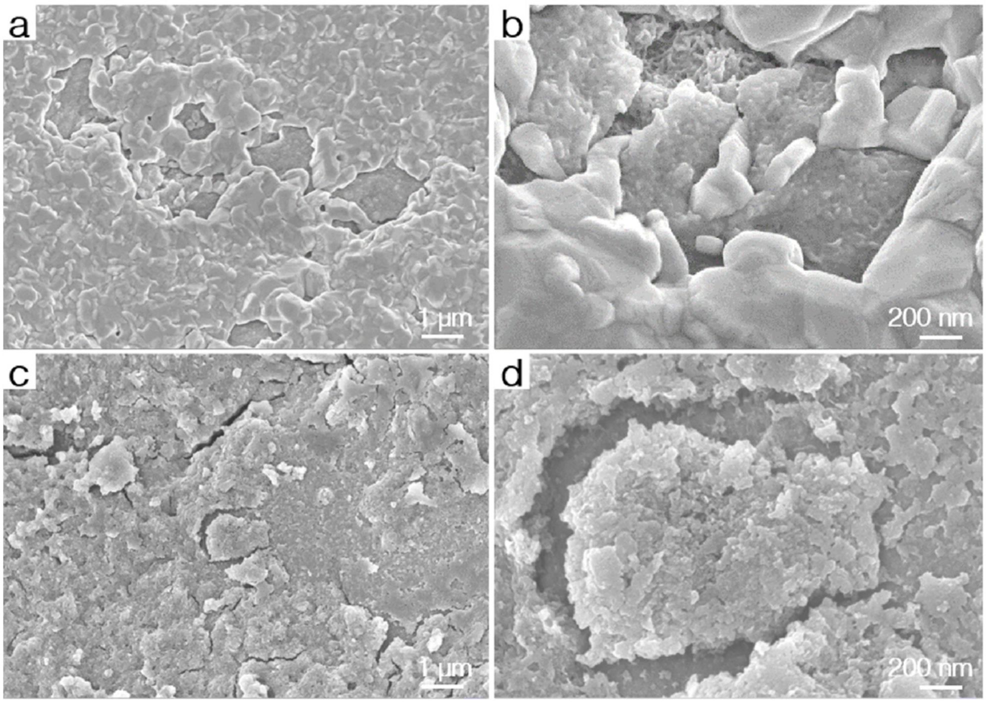

Figure S4. SEM images of Fenano (**a**,**b**) and Fe_2P (**c**,**d**).

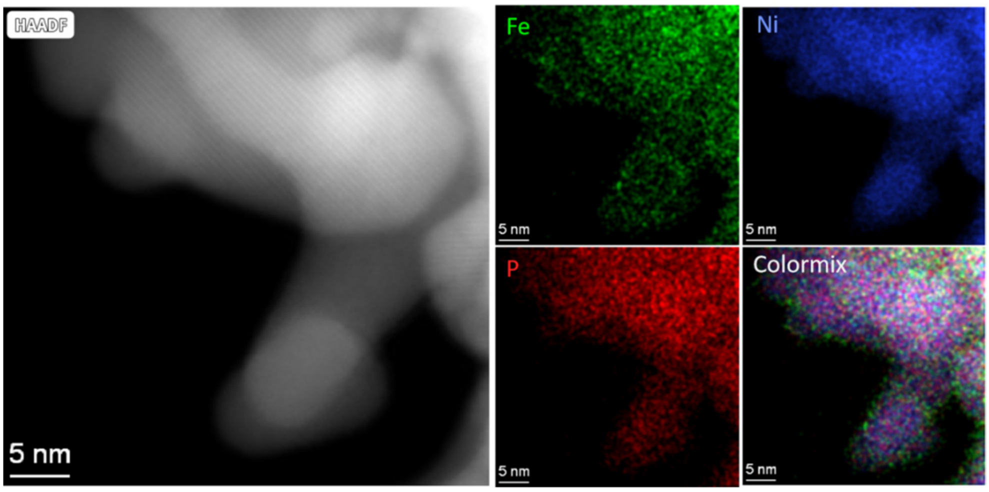

Figure S5. HAADF STEM and corresponding EDS mapping images of $Ni_2P@Fe_2P$.

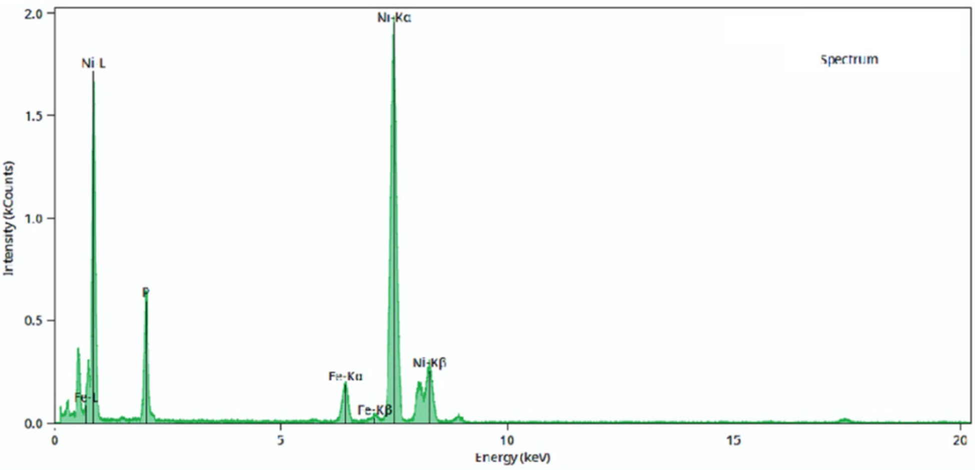

Z	Element	Family	Atomic Fraction (%)	Atomic Error (%)	Mass Fraction (%)	Mass Error (%)	Fit error (%)
15	P	K	18.87	3.84	10.97	1.33	0.62
26	Fe	K	6.52	1.59	6.84	1.23	0.25
28	Ni	K	74.61	18.72	82.19	15.64	0.14

Figure S6. EDS spectrum of as-prepared $Ni_2P@Fe_2P$.

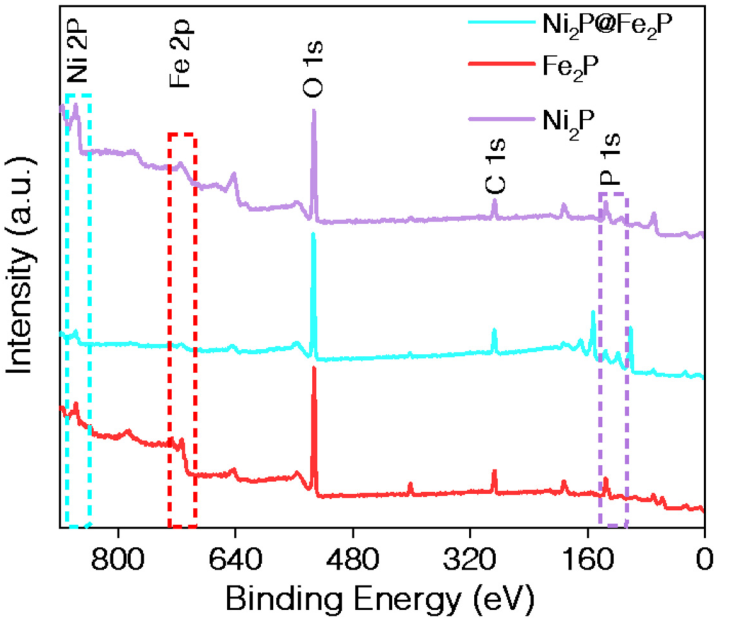

Figure S7. XPS survey spectra of the Ni_2P, $Ni_2P@Fe_2P$ and Fe_2P.

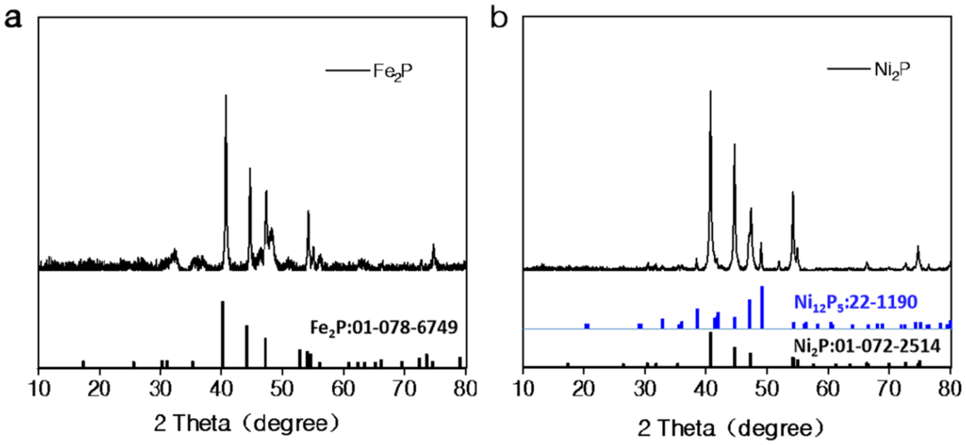

Figure S8. XRD patterns of (**a**) Fe_2P and (**b**) Ni_2P.

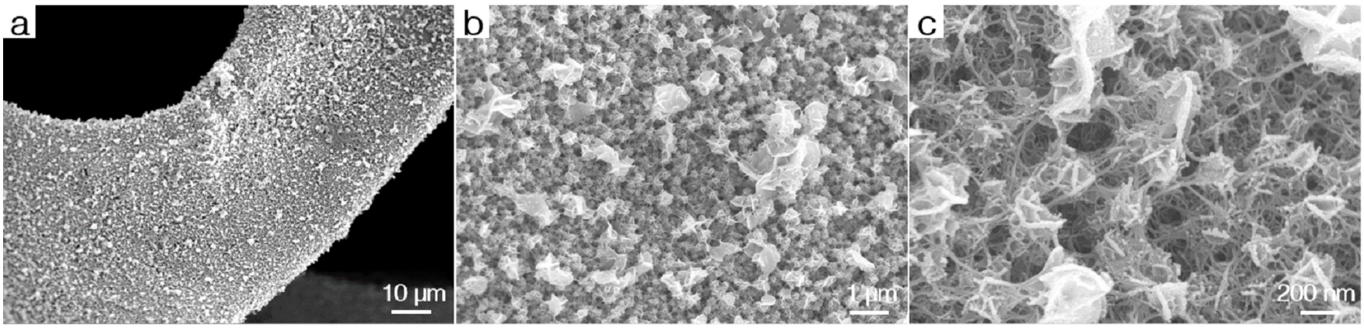

Figure S9. (a–c) SEM images of LDH with different magnifications.

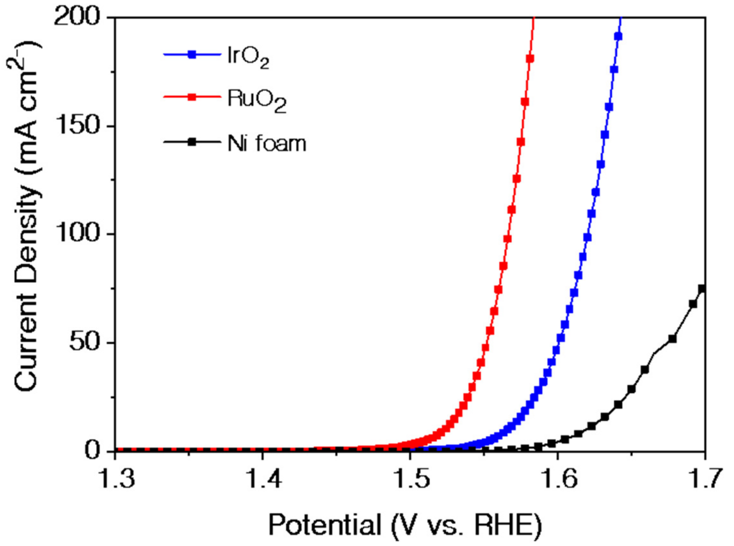

Figure S10. LSV curves of IrO_2, RuO_2 and Ni foam.

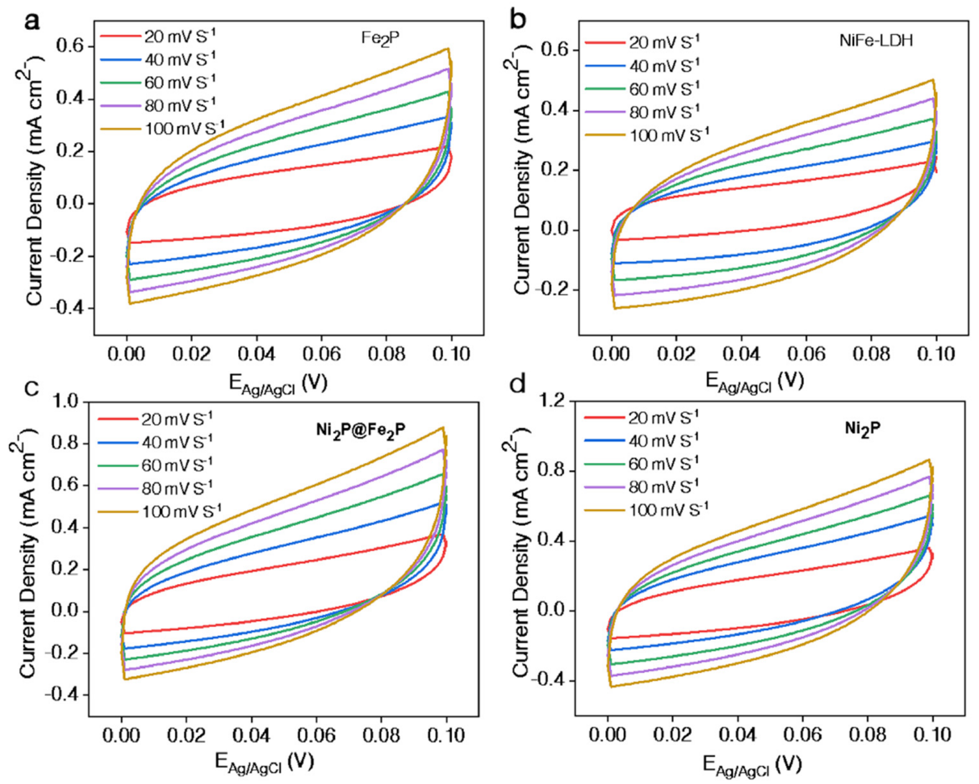

Figure S11. CV curves of (**a**) Fe_2P, (**b**) NiFe-LDH, (**c**) $Ni_2P@Fe_2P$, and (**d**) Ni_2P acquired at various scan rates.

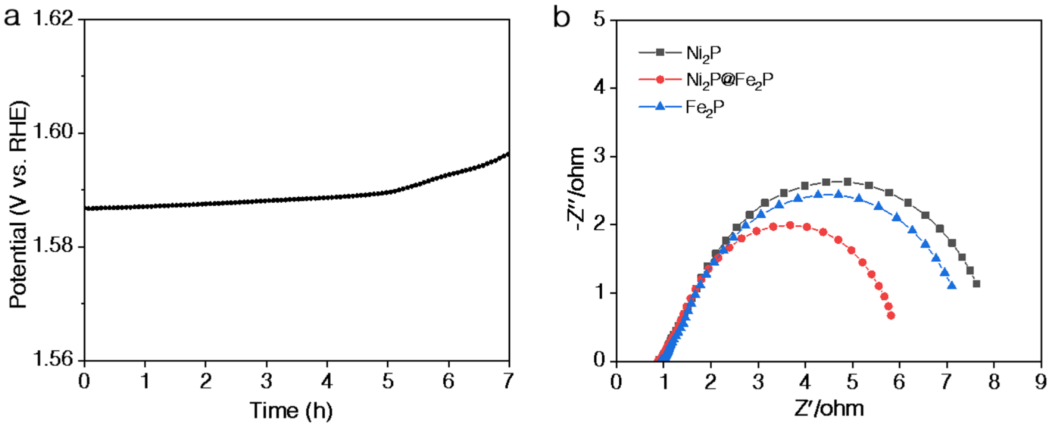

Figure S12. (**a**) Durability test of IrO_2 at 10 mA cm^{-2}. (**b**) Nyquist plots.

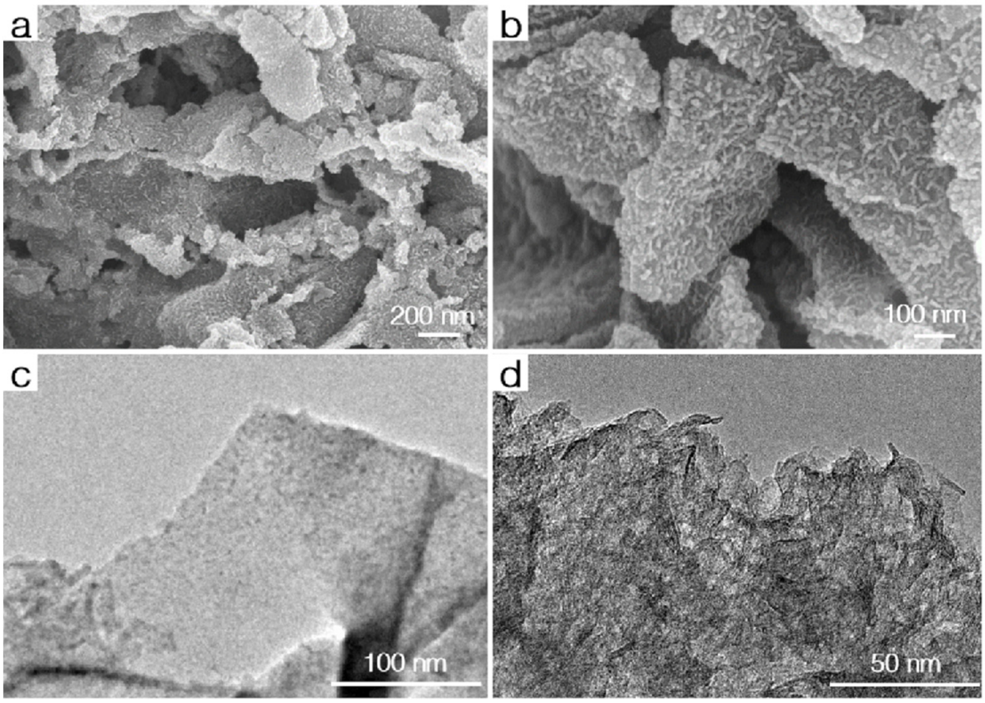

Figure S13. (**a**,**b**) SEM images of $Ni_2P@Fe_2P$ after LSV test. (**c**) TEM image of $Ni_2P@Fe_2P$. (**d**) TEM image of $Ni_2P@Fe_2P$ after LSV test.

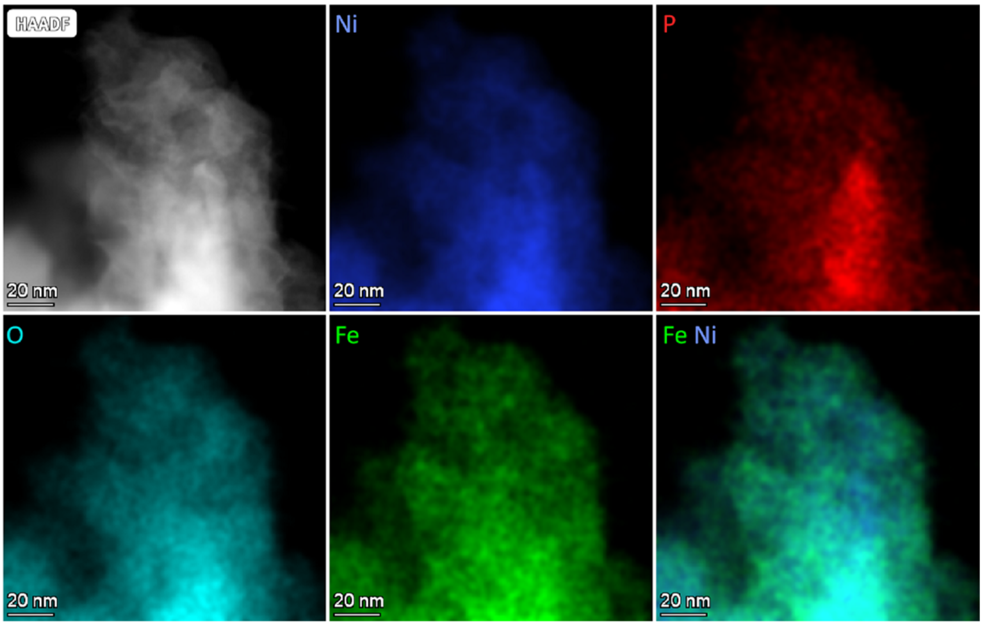

Figure S14. HAADF STEM and corresponding EDS mapping images of $Ni_2P@Fe_2P$ after LSV test.

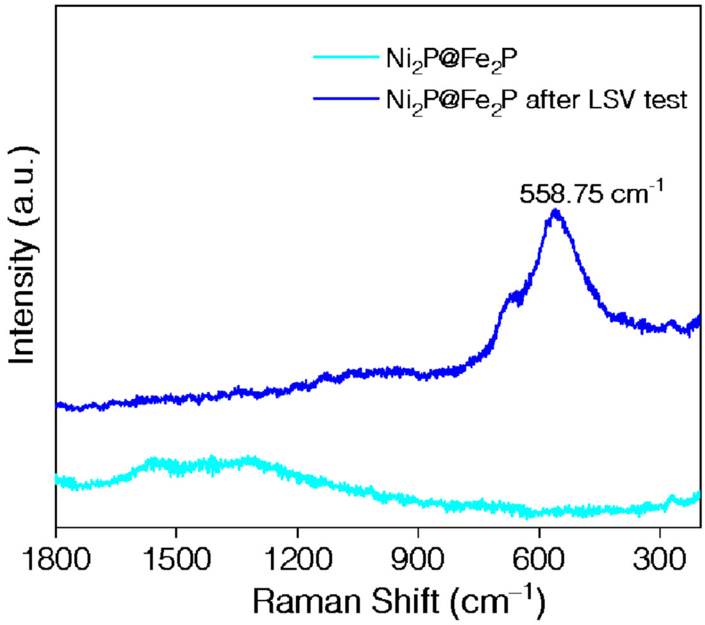

Figure S15. Raman spectra of $Ni_2P@Fe_2P$ before and after LSV test.

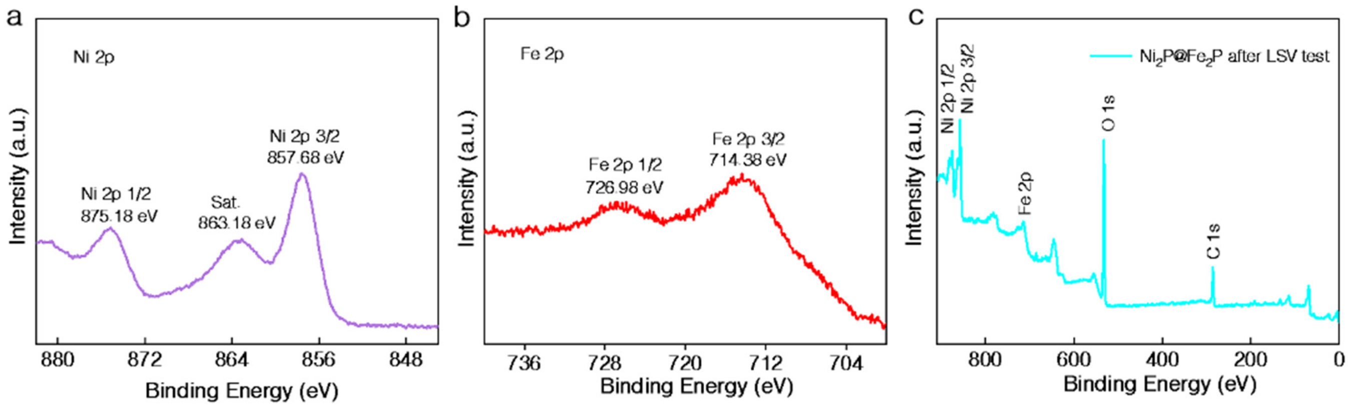

Figure S16. High-resolution XPS spectra of (**a**) Ni 2p, (**b**) Fe 2p and (**c**) $Ni_2P@Fe_2P$ after LSV test.

Table S1. Comparison of OER performances between $Ni_2P@Fe_2P$ electrode and recently reported electrocatalysts in alkaline solution.

Catalyst	Substrate	Overpotential (mV)	Tafel Slope (mV dec^{-1})	Stability	Ref.
$Ni_2P@Fe_2P$	[a]NF	210, 238, 247 @10, 50, 100 mA cm^{-2}	32.91	36 h@10, 50, 100 mA cm^{-2}	**This work**
NF@NiFe-LDH-1.5-4	NF	190@ 100 mA cm^{-2}	38.1	300 h@200 mA cm^{-2}	[2]
Ni@NiFe LDH	NF	269@100 mA cm^{-2}	66.3	24 h@10mA cm^{-2}	[3]
core-shell Ni-Fe-Cu	NF	220@100 mA cm^{-2}	33	20 h@10 mA cm^{-2}	[4]
NiFe-LDH	NF	460@100 mA cm^{-2}	27	24 h@20mA cm^{-2}	[5]
$NiFe(OH)_x$	NF	212@100 mA cm^{-2}	26.1	168 h@100 mA cm^{-2}	[6]
Amorphous (Fe-Ni) Co_x-OH/Ni_3S_2	NF	280@100 mA cm^{-2}	57	100 h@200 mA cm^{-2}	[7]
$CoFe(OH)_x$	NF	335@100 mA cm^{-2}	62.2	70h@10 mA cm^{-2}	[8]
Mo-NiOOH	NF	390@ 100 mA cm^{-2}	68	24 h@100mA cm^{-2}	[9]
$CoMoO_4@\gamma$-FeOOH	NF	279@100 mA cm^{-2}	46.7	36 h@10 mA cm^{-2}	[10]
FeOOH/CoO	NF	300@100 mA cm^{-2}	49.09	72 h@10 mA cm^{-2}	[11]
FeOOH/Cr-$NiCo_2O_4$	NF	268@100 mA cm^{-2}	31	10 h@20mA cm^{-2}	[12]
S-(Ni,Fe)OOH/NF	NF	281@100 mA cm^{-2}	48.9	100 h@100 mA cm^{-2}	[13]
FeOOH/NiFe LDHs	NF	290@100 mA cm^{-2}	NA	100 h@500 mA cm^{-2}	[14]
Zn-(Ni/FeOOH)	NF	269@100 mA cm^{-2}	33	1000 h@1000 mA cm^{-2}	[15]
FeOOH/CeO_2 HLNTs	NF	320@100 mA cm^{-2}	_	50 h@200 mA cm^{-2}	[16]

[a] NF; nickel foam.

References

1. Liang, J.; Gao, X.; Guo, B.; Ding, Y.; Yan, J.; Guo, Z.; Tse, E. C. M.; Liu, J. Ferrocene-Based Metal–Organic Framework Nanosheets as a Robust Oxygen Evolution Catalyst. Angew. Chem. Int. Ed. 2021, 60 (23), 12770–12774. https://doi.org/10.1002/anie.202101878.
2. Li, X.; Liu, C.; Fang, Z.; Xu, L.; Lu, C.; Hou, W. Ultrafast Room-Temperature Synthesis of Self-Supported NiFe-Layered Double Hydroxide as Large-Current–Density Oxygen Evolution Electrocatalyst. *Small* **2022**, *18* (2), 2104354. https://doi.org/10.1002/smll.202104354.
3. Cai, Z.; Bu, X.; Wang, P.; Su, W.; Wei, R.; Ho, J. C.; Yang, J.; Wang, X. Simple and Cost Effective Fabrication of 3D Porous Core–Shell Ni Nanochains@NiFe Layered Double Hydroxide Nanosheet Bifunctional Electrocatalysts for Overall Water Splitting. *J. Mater. Chem. A* **2019**, *7* (38), 21722–21729. https://doi.org/10.1039/C9TA07282A.
4. Zhang, P.; Li, L.; Nordlund, D.; Chen, H.; Fan, L.; Zhang, B.; Sheng, X.; Daniel, Q.; Sun, L. Dendritic Core-Shell Nickel-Iron-Copper Metal/Metal Oxide Electrode for Efficient Electrocatalytic Water Oxidation. *Nat. Commun.* **2018**, *9* (1), 381. https://doi.org/10.1038/s41467-017-02429-9.
5. Senthil, R. A.; Pan, J.; Yang, X.; Sun, Y. Nickel Foam-Supported NiFe Layered Double Hydroxides Nanoflakes Array as a Greatly Enhanced Electrocatalyst for Oxygen Evolution Reaction. *Int. J. Hydrog. Energy* **2018**, *43* (48), 21824–21834. https://doi.org/10.1016/j.ijhydene.2018.10.015.
6. Liu, X.; Gong, M.; Xiao, D.; Deng, S.; Liang, J.; Zhao, T.; Lu, Y.; Shen, T.; Zhang, J.; Wang, D. Turning Waste into Treasure: Regulating the Oxygen Corrosion on Fe Foam for Efficient Electrocatalysis. *Small* **2020**, *16* (24), 2000663. https://doi.org/10.1002/smll.202000663.
7. Che, Q.; Li, Q.; Chen, X.; Tan, Y.; Xu, X. Assembling Amorphous (Fe-Ni)Co -OH/Ni3S2 Nanohybrids with S-Vacancy and Interfacial Effects as an Ultra-Highly Efficient Electrocatalyst: Inner Investigation of Mechanism for Alkaline Water-to-Hydrogen/Oxygen Conversion. *Appl. Catal. B Environ.* **2020**, *263*, 118338. https://doi.org/10.1016/j.apcatb.2019.118338.
8. Zhu, G.; Li, X.; Liu, Y.; Mao, Y.; Liang, Z.; Ji, Z.; Shen, X.; Sun, J.; Cheng, X.; Mao, J. Scalable Surface Engineering of Commercial Metal Foams for Defect-Rich Hydroxides towards Improved Oxygen Evolution. *J. Mater. Chem. A* **2020**, *8* (25), 12603–12612. https://doi.org/10.1039/D0TA02858D.
9. Jin, Y.; Huang, S.; Yue, X.; Shu, C.; Shen, P. K. Highly Stable and Efficient Non-Precious Metal Electrocatalysts of Mo-Doped NiOOH Nanosheets for Oxygen Evolution Reaction. *Int. J. Hydrog. Energy* **2018**, *43* (27), 12140–12145. https://doi.org/10.1016/j.ijhydene.2018.04.181.
10. Song, H.; Li, J.; Sheng, G.; Zhang, Y.; Mohamad, A. A.; Luo, J.; Zhong, Z.; Shao, W. Construction of Core–Shell CoMoO4@γ-FeOOH Nanosheets for Efficient Oxygen Evolution Reaction. *Nanomaterials* **2022**, *12* (13), 2215. https://doi.org/10.3390/nano12132215.
11. Qiu, C.; He, S.; Wang, Y.; Wang, Q.; Zhao, C. Interfacial Engineering FeOOH/CoO Nanoneedle Array for Efficient Overall Water Splitting Driven by Solar Energy. *Chem. – Eur. J.* **2020**, *26* (18), 4120–4127. https://doi.org/10.1002/chem.201904352.
12. Liu, T.; Diao, P. Nickel Foam Supported Cr-Doped NiCo2O4/FeOOH Nanoneedle Arrays as a High-Performance Bifunctional Electrocatalyst for Overall Water Splitting. *Nano Res.* **2020**, *13* (12), 3299–3309. https://doi.org/10.1007/s12274-020-3006-3.
13. Yu, L.; Wu, L.; McElhenny, B.; Song, S.; Luo, D.; Zhang, F.; Yu, Y.; Chen, S.; Ren, Z. Ultrafast Room-Temperature Synthesis of Porous S-Doped Ni/Fe (Oxy)Hydroxide Electrodes for Oxygen Evolution Catalysis in Seawater Splitting. *Energy Environ. Sci.* **2020**, *13* (10), 3439–3446. https://doi.org/10.1039/D0EE00921K.
14. Chi, J.; Yu, H.; Jiang, G.; Jia, J.; Qin, B.; Yi, B.; Shao, Z. Construction of Orderly Hierarchical FeOOH/NiFe Layered Double Hydroxides Supported on Cobaltous Carbonate Hydroxide Nanowire Arrays for a Highly Efficient Oxygen Evolution Reaction. *J. Mater. Chem. A* **2018**, *6* (8), 3397–3401. https://doi.org/10.1039/C7TA10747A.

15. Zhang, X.; Yi, H.; Jin, M.; Lian, Q.; Huang, Y.; Ai, Z.; Huang, R.; Zuo, Z.; Tang, C.; Amini, A.; Jia, F.; Song, S.; Cheng, C. In Situ Reconstructed Zn Doped $Fe_xNi_{(1-x)}OOH$ Catalyst for Efficient and Ultrastable Oxygen Evolution Reaction at High Current Densities. *Small* **2022**, 2203710. https://doi.org/10.1002/smll.202203710.
16. Feng, J.-X.; Ye, S.-H.; Xu, H.; Tong, Y.-X.; Li, G.-R. Design and Synthesis of FeOOH/CeO_2 Heterolayered Nanotube Electrocatalysts for the Oxygen Evolution Reaction. *Adv. Mater.* **2016**, *28* (23), 4698–4703. https://doi.org/10.1002/adma.201600054.
